# GPU-Accelerated Compartmental Modeling Analysis of DCE-MRI Data from Glioblastoma Patients Treated with Bevacizumab

**DOI:** 10.1371/journal.pone.0118421

**Published:** 2015-03-18

**Authors:** Yu-Han H. Hsu, Ziyin Huang, Gregory Z. Ferl, Chee M. Ng

**Affiliations:** 1 Division of Clinical Pharmacology and Therapeutics, The Children's Hospital of Philadelphia, Philadelphia, PA, United States of America; 2 Early Development Pharmacokinetics and Pharmacodynamics, Genentech, South San Francisco, CA, United States of America; 3 Department of Pediatrics, Perelman School of Medicine, University of Pennsylvania, Philadelphia, PA, United States of America; Brighton and Sussex Medical School, UNITED KINGDOM

## Abstract

The compartment model analysis using medical imaging data is the well-established but extremely time consuming technique for quantifying the changes in microvascular physiology of targeted organs in clinical patients after antivascular therapies. In this paper, we present a first graphics processing unit-accelerated method for compartmental modeling of medical imaging data. Using this approach, we performed the analysis of dynamic contrast-enhanced magnetic resonance imaging data from bevacizumab-treated glioblastoma patients in less than one minute per slice without losing accuracy. This approach reduced the computation time by more than 120-fold comparing to a central processing unit-based method that performed the analogous analysis steps in serial and more than 17-fold comparing to the algorithm that optimized for central processing unit computation. The method developed in this study could be of significant utility in reducing the computational times required to assess tumor physiology from dynamic contrast-enhanced magnetic resonance imaging data in preclinical and clinical development of antivascular therapies and related fields.

## Introduction

Dynamic contrast-enhanced magnetic resonance imaging (DCE-MRI) is a noninvasive quantitative tool that allows analysis of tumor vascular characteristics that might change in response to drug treatments without the use of ionizing radiation. With DCE-MRI, vascular properties of tumors such as vascular permeability, rate of perfusion, and vascular leakage space can be quantified using standard MRI imaging system in routine clinical practice [1–3]. Therefore, this imaging technique is increasingly used to evaluate antivascular therapies and can also be applied to select drug doses for clinical studies, identify subpopulations enriched for clinical response, and predict patients’ benefits [4]. Numerous studies have utilized DCE-MRI as a quantitative imaging biomarker to guide preclinical and early clinical development of antiangiogenic agents, including anti-VEGF (vascular endothelial growth factor) antibodies such as bevacizumab (Avastin), receptor tyrosine kinase inhibitors, and vascular disrupting agents such as 5,6-dimethylxanthenone-4-acetic acid (DMXAA) [1, 4, 5].

Several analysis methods have been developed to quantify changes in microvascular physiology using DCE-MRI data [6–9]. Compartmental models such as the Tofts and the extended Tofts models are commonly used for kinetic analysis of imaging data and, when using an appropriate model structure, are the golden standards for quantification of DCE-MRI data [10, 11]. The analysis process involves using numerical minimization algorithms to fit the mathematical models to the observed contrast agent concentration-time profiles in blood and tissue and deriving model parameters that can estimate physiological properties. For instance, two tumor properties, fractional interstitial volume (*v*_*e*_) and fractional transfer rate (*K* ^*trans*^), are commonly derived and used to assess drug effects on tumor. While this kinetic modeling approach to analyze DCE-MRI data is straightforward in concept, it is often very time consuming and computationally expensive, as model fitting has to be performed separately on each voxel in a DCE-MRI image, requiring tens of thousands of minimization function calls. When a dataset contains multiple DCE-MRI scans from many patients, with multiple slices in each scan, the analysis time cost may become a significant burden in a fast-paced clinical or research environment.

The graphics processing unit (GPU) is a dedicated numerical processor that has evolved from a highly specialized graphics processor to a versatile, highly programmable and energy efficient architecture for scientific computing [12–14]. Several of the world’s fastest supercomputers, including Titan at Oak Ridge National Laboratory and Piz Daint at Swiss National Supercomputing Centre, are partly powered by GPU-computing technology [15]. Compared to standard central processing unit (CPU), current GPU has hundreds of numerical processor cores on a single chip and can be programmed to perform many numerical operations simultaneously to achieve extremely high arithmetic intensity for complex numerical analysis. Because of this unique architecture, running computations on GPU can be significantly faster than on CPU when the computations can be parallelized and distributed to GPU’s numerous processor cores. GPU parallel computing has been used in various medical imaging applications for faster processing [16–18]. For DCE-MRI reconstruction and analysis, since each voxel in a DCE-MRI image is normally treated as an independent element during compartmental modeling, the process is perfectly suited for parallel implementations on the GPU. Recently, we successfully used GPU to accelerate the performance of relatively simple model-independent nonparametric method in the analysis of clinical DCE-MRI data [19]. However, to our best knowledge, there is no published study or report of using GPU to improve the performance of standard compartmental model method in DCE-MRI data analysis. Therefore, in this article, we present the first GPU-accelerated compartmental modeling method for DCE-MRI data analysis that can drastically reduce the amount of time required to evaluate changes in microvascular physiology in clinical patients before and after antivascular therapies.

## Methods

### Compartmental Model for Describing the Blood and Tumor Concentration-Time Profiles of Contrast Agent

**Fig 1:**
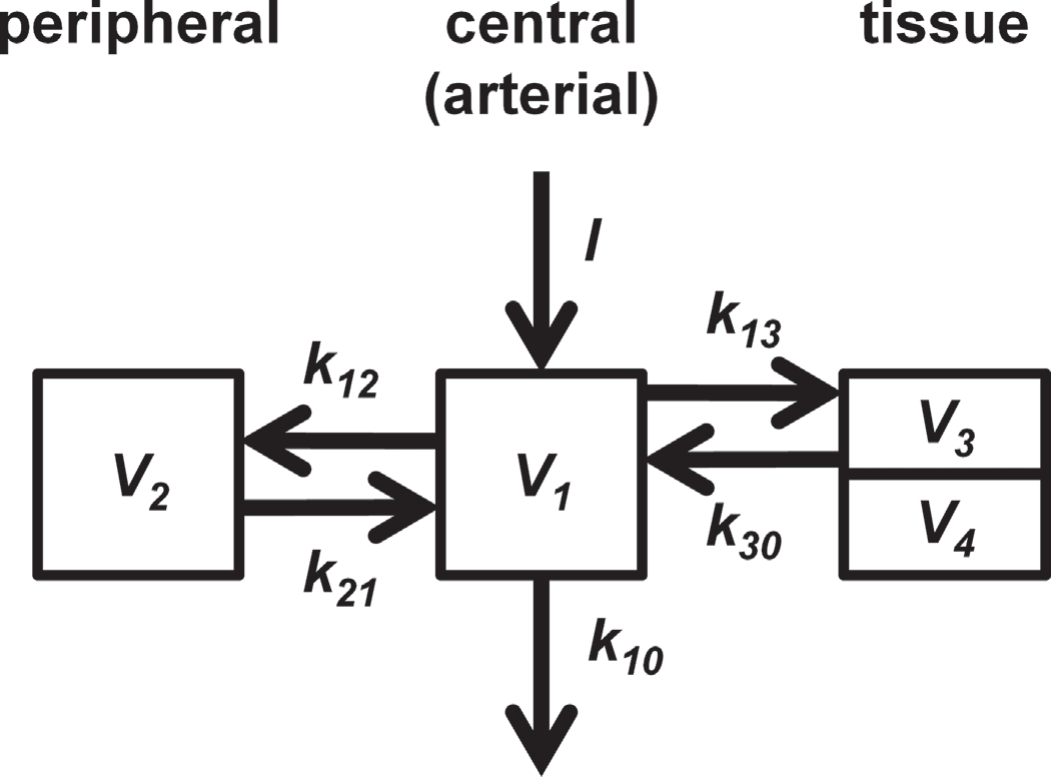
Compartment models for Gd-DTPA kinetics. The schematic shows the kinetics relationship between the tumor tissue (right box) and the remainder of the body (center and left box). Drug input (*I*) goes into arterial blood in compartment 1 (center box, volume: *V*_*1*_), which exchanges with a general peripheral compartment 2 (left box, volume: *V*_*2*_) via fractional clearances *k*_*12*_ and *k*_*21*_ and eliminates drug via *k*_*10*_. The tumor (and other brain tissue) is represented by 2 compartments: compartment 3 (top of right box, volume: *V*_*3*_) exchanges with compartment 1 via *k*_*13*_ and *k*_*30*_; compartment 4 (bottom of right box, volume: *V*_*4*_) exchanges with compartment 1 so rapidly that its drug concentration is practically the same as that of compartment 1.

The complete compartmental model used to describe the contrast agent kinetics in each DCE-MRI scan is shown in Fig. 1. A linear two-compartment model was used to represent systematic contrast agent kinetics (Fig. 1, compartments 1 and 2), thus the contrast agent concentration-time course in arterial blood (compartment 1) can be described by a bi-exponential decay equation [20–22]:

$$C_{1}\left(t\right)=\frac{R_{0}}{V_{1}}[\frac{(1-e^{\lambda_{1}\tau })(k_{21}-\lambda_{1})e^{-\lambda_{1}(t-t_{0}-t_{lag})}}{-\lambda_{1}(\lambda_{2}-\lambda_{1})}+\frac{(1-e^{\lambda_{2}\tau })(k_{21}-\lambda_{2})e^{-\lambda_{2}(t-t_{0}-t_{lag})}}{-\lambda_{2}(\lambda_{1}-\lambda_{2})}]$$ (1)

Where *R*_0_ is zero-order infusion rate; *V*_1_ is the volume of compartment 1; *t*_0_ is the starting time of infusion; *t*_*lag*_ is lag time before infusion starts to affect concentration observation; *τ* is the apparent elapsed time of infusion: *τ* = 0 for *t* ≤ *t*_0_ + *t*_*lag*_, *τ* = *t* – *t*_0_ − *t*_*lag*_ for *t*_0_ + *t*_*lag*_ < *t* ≤ *t*_1_ + *t*_*lag*_ (*t*_1_ is the time at the end of infusion), and *τ* = *t*_1_ – *t*_0_ for *t* > *t*_1_ + *t*_*lag*_; and $\lambda_{1,2}=\frac{1}{2}\left(k_{10}+k_{12}+k_{21}\pm \sqrt{{\left(k_{10}+k_{12}+k_{21}\right)}^{2}-4k_{10}k_{21}}\right)$ [20]. As shown in Fig. 1, *k*_10_, *k*_12_, and *k*_21_ are fractional clearances between the indicated compartments.

The extended Tofts model was used to depict contrast agent kinetics in tumor and other brain tissue. Since tissue consists of both extravascular extracellular space (EES) and a blood plasma volume, its total contrast agent concentration at time *t* is [21]:

$$C_{tissue}\left(t\right)=v_{e}C_{e}\left(t\right)+v_{p}C_{p}\left(t\right)$$ (2)

Where *C*_*e*_(*t*) and *C*_*p*_(*t*) are the contrast agent concentrations in EES and in blood plasma, respectively, and *v*_*e*_ and *v*_*p*_ are their corresponding volumes per unit volume of tissue. The EES and blood plasma compartments correspond to compartments 3 and 4, respectively, in Fig. 1. The EES (compartment 3) concentration-time course, *C*_*e*_(*t*), can be described by [20, 23, 24]:

$$\begin{array}{r} C_{3}\left(t\right)=k_{30}\frac{R_{0}}{V_{1}}[\frac{(1-e^{\lambda_{1}\tau })(k_{21}-\lambda_{1})e^{-\lambda_{1}(t-t_{0}-t_{lag})}}{-\lambda_{1}(\lambda_{2}-\lambda_{1})(k_{30}-\lambda_{1})}\\ +\frac{(1-e^{\lambda_{2}\tau })(k_{21}-\lambda_{2})e^{-\lambda_{2}(t-t_{0}-t_{lag})}}{-\lambda_{2}(\lambda_{1}-\lambda_{2})(k_{30}-\lambda_{2})}\\ +\frac{(1-e^{k_{30}\tau })(k_{21}-k_{30})e^{-k_{30}(t-t_{0}-t_{lag})}}{-k_{30}(\lambda_{1}-k_{30})(\lambda_{2}-k_{30})}] \end{array}$$ (3)

(the variables have same definitions as in Equation 1; *k*_30_ is used instead of *k*_31_ to indicate that contrast agent transfer from compartment 3 back into compartment 1 is ignored when defining *C*_1_(*t*)). The blood plasma (compartment 4) concentration-time course, *C*_*p*_(*t*), can be estimated by the *C*_1_(*t*) model in Equation 1, since the relatively fast blood exchange between compartments 1 and 4 would make their concentrations practically indistinguishable. It is important to note that *k*_30_ = *K* ^*trans*^ / *v*_*e*_, where *K* ^*trans*^ is the volume transfer coefficient between arterial blood and EES. Thus the extended Tofts model relates tissue concentration data to both *v*_*e*_ and *K* ^*trans*^, two important parameters that are commonly used to assess tumor physiology and vascular function in clinical studies. These parameters are obtained by using the nonlinear optimization algorithm such as Newton and Quasi-Newton methods to fit ESS compartmental model (Equation 3) to the observed contrast agent concentration-time course in individual voxel of human tissue image scans. Because each image scan of the human tissue contains many thousands voxels, this particular step of repeatedly fitting the ESS model to observed contrast agent concentration-time courses requires tens of thousands of minimization function calls and therefore, is extremely time consuming and computationally expensive to perform.

### A GPU-based Implementation

Data parallelism is an essential requirement for an algorithm to benefit from GPU execution. The most computational step of the compartment analysis approach is the fitting of the ESS model to the observed contrast agent concentration-time course of many thousands of voxel in each human tissue image scan. However, this particular computation step may be formulated in a GPU-friendly data parallel manner as it mainly consist of voxel-wise computations.

#### Analysis Flow of the GPU-accelerated compartmental modeling approach

**Fig 2:**
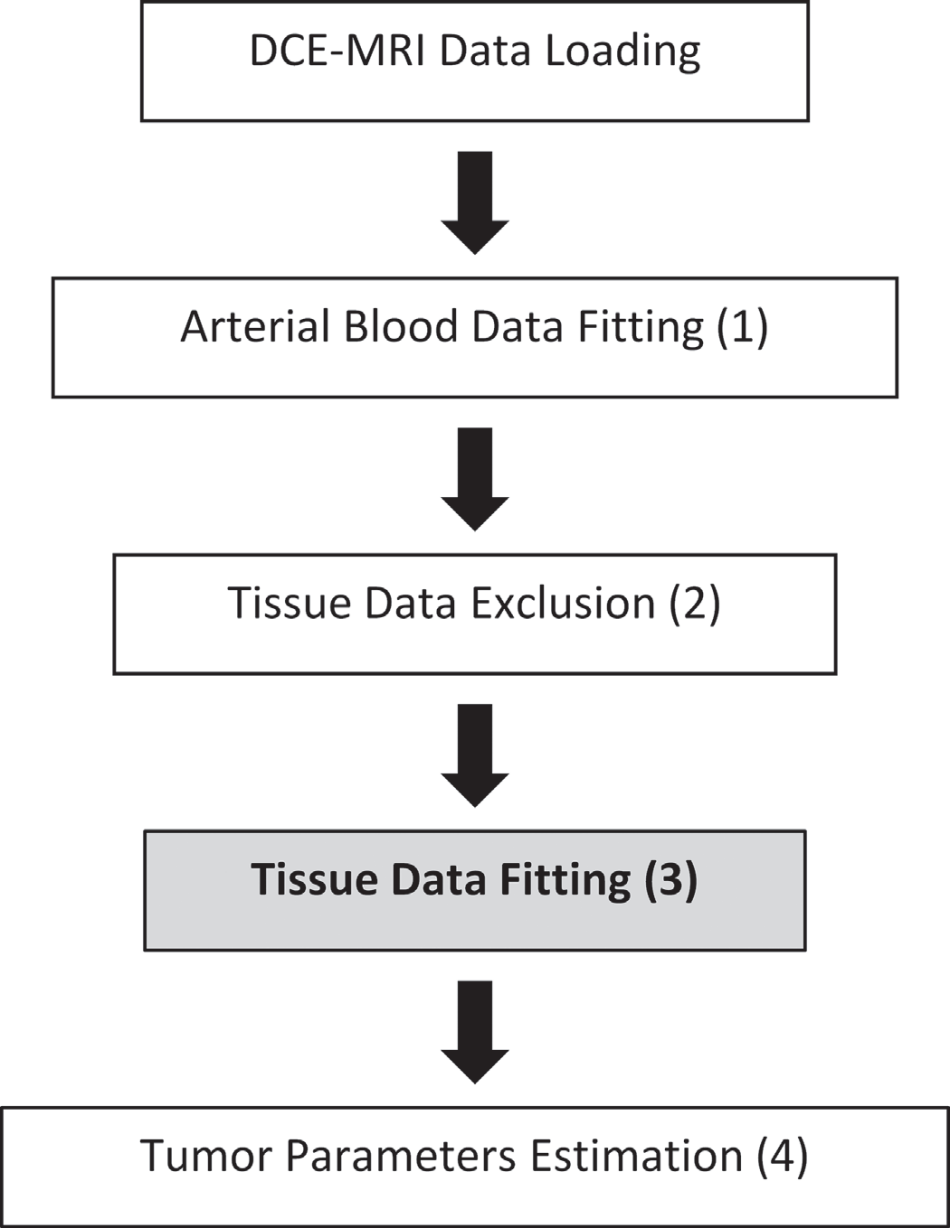
Flow chart of the analysis steps executed by the MATLAB script. Step done on the GPU is in bold font.

Fig. 2 summarizes the analysis flow of the GPU-accelerated compartmental modeling program which developed using MATLAB (The MathWorks, Inc., Natick, MA) and MATLAB-compatible GPU computing toolbox Jacket v2.0 (Accelereyes Inc., Atlanta, GA) which became part of MATLAB parallel computing toolbox, and each step is described in more detail below, and the MATLAB function code of the GPU-accelerated compartment modeling program was included as the supplementary materials (S1–S3 Programs).

*1*. *Arterial Blood Data Fitting*. After DCE-MRI data is loaded by the MATLAB script, the linear two-compartment model described by Equation 1 is fit to the arterial contrast agent concentration (***C***_***v***_) data by using the CPU-based Nelder-Mead algorithm to minimize the least squares objective function:

$$f\left(p\right)=\sum_{i=1}^{n_{f}}{\left(c_{i}-{\hat{c}}_{i}\left(p\right)\right)}^{2}$$ (4)

Where *p* is the set of model parameters to be estimated, including *R*_0_/*V*_1_ (treated as one parameter), *k*_10_, *k*_12_, *k*_21_, and *t*_*lag*_; *c*_*i*_ is the observed arterial concentration at each time point; and ${\hat{c}}_{i}\left(p\right)$ is the corresponding concentration value predicted by Equation 1 with the given *p* values. Inputs to the Nelder-Mead algorithm, such as initial estimates and boundaries of the model parameters, the number of iterations (300), and the convergence threshold (1e-5), are set based on past literature and preliminary analysis results. After ***C***_***v***_ data fitting is completed, fitted parameter values for *R*_0_/*V*_1_, *k*_10_, *k*_12_, and *k*_21_ are stored and used for fitting tissue data in Step 3 (Fig. 2).

*2*. *Tissue Data Exclusion*. Before tissue data fitting is performed, the tissue contrast agent concentrations (***C***_***T***_) matrix is screened to exclude any voxels whose data contain: a) missing values, b) only negative values, or c) values that exceed a maximum threshold (defined based on fitted arterial data). This procedure removes extremely noisy data, often from voxels outside of the brain, and saves unnecessary computational time in the following analysis steps. For the DCE-MRI data analyzed in this study, more than 50% of the voxels in the field of view (FOV) can be excluded from further analysis in this step.

*3*. *Tissue Data Fitting*. After noisy voxels are removed, GPU-accelerated Nelder-Mead minimization of the objective function in Equation 4 is performed on each remaining voxel’s ***C***_***T***_ data. In this case, *c*_*i*_ is the observed tissue concentration in a single voxel at each time point and ${\hat{c}}_{i}\left(p\right)$ is calculated from the extended Tofts model (Equation 2). The set of model parameters to be estimated (*p*) are *v*_*e*_, *k*_30_, *v*_*b*_, and *t*_*lag*_. Other parameters, including *R*_0_/*V*_1_, *k*_10_, *k*_12_, and *k*_21_, are set to the values derived from arterial data fitting in Step 1 (Fig. 2).

*4*. *Tumor Parameters Estimation*. After all tissue data fitting is completed, the resulting parameter estimates are generated by the MATLAB script. The tumor parameters *v*_*e*_ and *K* ^*trans*^ can be derived from results of the ***ROI*** voxels (using *k*_30_ = *K* ^*trans*^ / *v*_*e*_) and can be visualized in heat maps after adjusting for extreme outliers. Median ***ROI*** *v*_*e*_ and *K* ^*trans*^ values can be calculated to compare tumor physiology and function in the scans acquired before and after bevacizumab treatment.

#### A GPU-accelerated Nelder-Mead simplex-based minimization algorithm

*1*. *Clinical Imaging Study and Image Processing*. The DCE-MRI data analyzed in this study were collected in a phase II clinical trial of bevacizumab in patients with grade III-IV glioma that was approved by the Duke Institutional Review Board and informed consent was obtained from every patients [9, 25]. DCE-MRI scans were obtained from the patients one day before and one day after bevacizumab treatment, using gadolinium-diethylene triamine pentaacetic acid (Gd-DTPA) as the contrast agent. Details of the DCE-MRI procedure and preliminary image processing were described in previous studies [7, 9, 19].

*2*. *Model Fitting Minimization Algorithm*. A Nelder-Mead simplex-based minimization algorithm was developed and used to fit the concentration-time models to the DCE-MRI blood and tissue data and to estimate the model parameters. The classic Nelder-Mead method [26] is often used to solve unconstrained minimization problem in which the goal is to minimize a function of *n* variables. In brief, the method constructs a simplex with *n+1* vertices in the search space (where each vertex represents a set of variable values), then performs a series of transformations on the simplex to decrease the function values of its vertices until convergence is reached (i.e. the vertices or their corresponding function values become close enough).

The minimization algorithm implemented in this study was modified to add an extra step to the classic Nelder-Mead method so that it can be used to solve constrained optimization problem [27]. More specifically, the algorithm takes in a set of initial estimates (*x*_0_) for the function variables along with lower (*lb*) and upper (*ub*) bound restrictions on the variable values, then performs an arcsine transformation:

$$x_{u}=\arcsin\left(\frac{2(x_{0}-lb)}{ub-lb}-1\right)$$ (5)

to convert *x*_0_ into its counterpart (*x*_*u*_) in an unconstrained search space. After this transformation, the normal Nelder-Mead process is carried out to find a temporary solution in the unconstrained space, then the temporary solution is reversely transformed to produce a final solution that confines to the original boundary restrictions.

The code in this constrained Nelder-Mead algorithm was also modified extensively to remove and/or replace branching (e.g. if-else and switch case statements) and conditional termination, since these operations are computationally expensive and cannot run properly in parallel on the GPU without major modifications. Thus while a normal Nelder-Mead function would terminate whenever it reaches convergence or a pre-determined iteration number, the Nelder-Mead algorithm developed in this analysis runs a pre-determined number of iterations in two sequential steps. Initially, a first and short minimization process is performed on all voxels with a pre-defined number of iterations regardless of convergence. After the first minimization process is completed, voxels that have reached convergence are removed from subsequent analysis. Then a second minimization process is performed on the remaining unconverged voxels using the best parameter estimates obtained in the first step as the initial estimates. This multiple-step approach prevents the branching problem associated with early conditional termination of the minimization algorithm on GPU, which can produce unreliable results and decrease the efficiency of GPU computation. Testing on simulated data showed that these changes in code structure do not significantly alter minimization results.

To evaluate the performance of the GPU-accelerated kinetic analysis program developed in this study, both the algorithm that performs the identical analysis on the CPU (constrained Nelder-Mead algorithm with branching and conditional termination removed) and the algorithm optimized for CPU calculation (constrained Nelder-Mead algorithm without branching and conditional termination removed) were implemented to generate results for comparison purposes.

The following data is used to perform voxel-wise kinetic analysis of each DCE-MRI slice: a) ***Time***, a 1 × *n*_*f*_ vector that indicates data acquisition time points, where *n*_*f*_ is the total number of image frames, b) ***C***_***v***_, a 1 × *n*_*f*_ vector that describes the vascular contrast agent concentrations in arterial blood, c) ***C***_***T***_, an *n*_*x*_ × *n*_*y*_ × *n*_*f*_ matrix that describes the contrast agent concentrations in tumor and other brain tissue, where *n*_*x*_ × *n*_*y*_ are the dimensions of the FOV, and d) ***ROI*** (region of interest), an *n*_*x*_ × *n*_*y*_ matrix that indicates which voxels in the FOV are in the tumor region (*n*_*x*_ × *n*_*y*_ for both ***C***_***T***_ and ***ROI*** is 256 x 256, since FOV has a dimension of 256 x 256 mm) [19].

All analyses were executed on a 64-bit Windows 7 (Microsoft Corporation, Redmond, WA) desktop computer with Intel Xeon X5690 CPU (Intel Corporation, Santa Clara, CA) and a NVIDIA Tesla C2070 GPU card (NVIDIA, Santa Clara, CA) that contains 448 numerical processor cores and 6 GB onboard SDRAM memory.

## Results

### Estimations of *v*_*e*_ and *K* ^*trans*^

**Fig 3:**
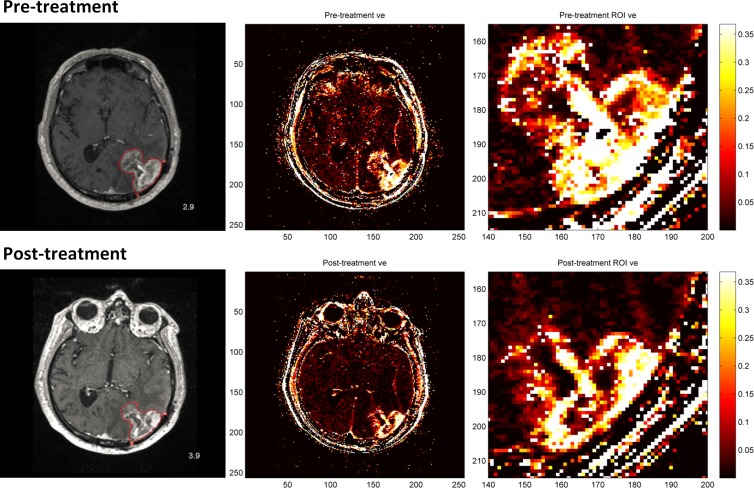
The *v*_e_ results of a single axial slice from DCE-MRI brain scans. The top row is from a pre-treatment scan and the bottom row is from a post-treatment scan. The left panel shows the original slice images with the tumor tissue (*ROI*) circled in red, the middle panel displays *v*_*e*_ values derived from the slices, and the right panel shows close-up *v*_*e*_ heat maps of the *ROI*.

DCE-MRI scans taken one day before (pre-treatment) and one day after (post-treatment) administration of a single 10mg/kg bevacizumab dose in a glioblastoma patient were used to assess the performance of the GPU-accelerated kinetic analysis method implemented in this study. The *v*_*e*_ values derived from a single axial slice within each scan are displayed as heat maps in Fig. 3. The tumor tissue (*ROI*) is clearly visible in the lower right corner of the brain (left and middle panels), and there is an overall decrease in tumor *v*_*e*_ intensity after treatment. The *K* ^*trans*^ heat maps also display similar patterns as the *v*_*e*_ results (S1 Fig.). Across all *ROI* voxels in this slice, median *v*_*e*_ values (unitless) are 0.242 in the pre-treatment scan and 0.103 in the post-treatment scan, while median *K* ^*trans*^ values are 0.106 and 0.0943 min^-1^ before and after treatment, respectively (Table 1). These results indicate a 57.4% decrease in tumor *v*_*e*_ and an 11.4% decrease in tumor *K* ^*trans*^ following treatment. These numbers are comparable with results from past studies [9, 10], and also agree with the expectation that tumor *v*_*e*_ and *K* ^*trans*^ should decrease after antiangiogenic treatment. Furthermore, results generated from the GPU script were compared to results of a CPU script that performs the same analysis steps in serial to verify that parallelization on the GPU does not alter the outcome of the analysis method: most of the GPU and CPU results (99.9%) are very similar and show less than 5% difference.

**Table 1:** Median *v*_*e*_ (unitless) and *K* ^*trans*^ (min^−1^) values in the *ROI* for data considered in Fig. 3.

Parameter	Pre-treatment	Post-treatment	% change^1^
***v***_***e***_	0.242	0.103	−57.4
***K*** ^***trans***^	0.106	0.0943	−11.4

^1^% change: the percentage change in pre- and post-treatment values.

### Time Performance

The time performance of the GPU-accelerated compartmental modeling analysis method is significantly better than a CPU-based method that performs the same analysis steps in serial. The time results shown here came from timing the analyses of 16 different DCE-MRI slices. The statistical analyses of the performances are shown in Table 2. On average, using a single NVIDIA C2070 GPU card, the GPU-based method could analyze a DCE-MRI slice in ∼44 seconds, which is approximately 124 times faster than the CPU method. Taking the number of voxels included in each analysis into account, the GPU script could analyze ∼593 voxels per second, while the CPU script analyzed fewer than 5 voxels per second. Even when compared to an optimized CPU method that uses a more efficient Nelder-Mead algorithm (with conditional branching and termination check), the GPU method still perform more than 17 times faster.

**Table 2:** Time performance of analyzing the GPU-accelerated method developed in this study versus a CPU-based methods with the same analysis steps.

Method	# of seconds per slice (avg. ± std. dev.^1^)	# of voxels per second (avg. ± std. dev. ^1^)	Compared to GPU
**GPU**	44.20 ± 1.30	593.72 ± 13.01	1x
**CPU (same algorithm structure with GPU)**	5468.70 ± 218.82	4.80 ± 0.05	∼124x slower
**CPU (optimized algorithm structure for CPU)**	761.84 ± 34.32	34.46 ± 0.49	∼17x slower

^1^ avg. ± std. dev.: average ± standard deviation

## Discussion

In this study, we utilized the power of parallel computing and developed a GPU-accelerated compartmental modeling method for analyzing clinical DCE-MRI data. Executed with a single GPU card, our method offers a 124-fold speedup and yields similar parameter estimations compared to a CPU-based method that contains analogous serial code. This article is the first published report of a GPU-based compartmental modeling method of DCE-MRI data, and proposes an efficient way to assess important tumor physiological properties before and after antivascular therapies in clinical patients.

The method described in this study was specifically designed to utilize the power of GPU-computing technology in order to analyze DCE-MRI data efficiently, thus many choices we made during method development reflect this main purpose. In the early phase of development, the computational times of all analysis steps were assessed and evaluated for the feasibility of implementing the program code in a GPU-computing platform. The tissue data fitting process for each DCE-MRI voxel was identified as the most computationally intensive and rate-limiting step of our method because data from each voxel has to be analyzed separately and the Nelder-Mead minimization process has to be performed many times during this step. Consequently, we focused on parallelizing this step on the GPU in order to improve the computing efficiency of our method.

First, we chose to implement a Nelder-Mead algorithm that can simultaneously perform minimization on tissue data from many voxels on the GPU. This simplex-based direct search algorithm was selected over other minimization algorithms because its operations are deterministic and its implementation is relatively straightforward on the GPU. Other deterministic minimization algorithms such as the Gauss-Newton method were considered during early development, but they involve complicated numerical derivative and hessian matrix calculations that are difficult to implement properly or execute efficiently for DCE-MRI analysis on the GPU. A probabilistic search minimization algorithm, simulated annealing, was also tested, but this method produced inconsistent results due to its stochastic nature and would require a large number of iterations to yield more satisfactory results. In comparison, the Nelder-Mead algorithm is a relatively simple derivative-free minimization method that requires a relatively small number of iterations and computations, so it is suited for GPU parallel computing and its results are easily reproducible during testing. After selecting the Nelder-Mead algorithm as the minimization function in our DEC-MRI analysis method, we modified it to optimize its execution on the GPU. In particular, our GPU Nelder-Mead function was designed to run a pre-determined number of iterations on all voxels analyzed in parallel. This design is necessary because conditional termination could not be properly implemented in our Nelder-Mead function: when voxels are analyzed in a single kernel pass on the GPU, if a single voxel’s minimization process terminates due to convergence, other voxels’ processes would also terminate prematurely, thus producing unrealistic results.

During preliminary analysis, we noted that while the majority of the DCE-MRI voxels reach convergence relatively early in the minimization process, and only a small group of voxels requires many more iterations. For instance, for the data shown in Fig. 3, more than 50% of the voxels converge with fewer than 100 iterations during analysis. Therefore, it is not very time efficient to set a large number of iterations for all voxels while running the Nelder-Mead algorithm. We decided to break up the minimization process into two steps: in the first step, a small number of iterations are performed on all voxels; in the second step, more iterations are performed only on voxels that have not achieved convergence in the first step. Several iteration combinations for the first and second minimization steps were tested during preliminary analysis, and a combination (150 and 200) that had the best time performance for our dataset was used in the final minimization process. Using this design, we were able to further improve the best time performance of the entire GPU-accelerated kinetic analysis script from more than a minute to around 40 seconds.

The Compute Unified Device Architecture (CUDA) is a programming platform developed by NVIDIA for general-purpose computing on CUDA-compatible NVIDIA graphics processor cards. The development of CUDA allows scientific researchers to take advantage of GPU’s parallel computing power for complex numerical computations without using any complicated graphics-specific application programming interface [28]. The GPU-computing feature described in this study was implemented in MATLAB using Jacket (now part of its parallel computing toolbox), a numerical computing platform which compiles MATLAB code into CUDA-compatible code that runs on CUDA-enabled GPUs. The parallel analysis steps utilizes the GFOR (GPU for) loop construct implemented in Jacket to simultaneously launch and perform the minimization process on many voxels’ data on the GPU [29]. Developing the algorithm using MATLAB is convenient because it allows scientific researchers to rapidly develop prototype programs for GPU-accelerated data analysis. Furthermore, the developed GPU code can easily be converted to standalone, license-free and MATLAB-independent executables for deployment to larger user bases in the scientific community. While the MATLAB script of our GPU-based method performs well compared to CPU methods, potential time performance improvement may be achieved by using a lower-level GPU-computing platform (e.g. C for CUDA) to implement the method. Also, while the results shown in our study were obtained using only a single GPU card, it is expected that our program can be even more efficient in a multi-GPU computing environment. Further study is ongoing to assess the performance improvement our method can achieve using multiple GPU cards.

## Conclusion

The findings of this study suggest that GPU-accelerated method in this study can be a practical and efficient alternative to previously reported DCE-MRI compartmental modeling analysis methods. The method significantly reduces the computational time required to obtain important tumor physiological properties, allowing researchers to quickly identify potential regions of interest when analyzing a scan and assess presence or absence of a treatment effect when comparing pre- and post- treatment scans. Furthermore, although this paper focuses on applying the GPU-accelerated method to brain tumor data and uses the extended Tofts model for analysis, the method can easily be extended to analyze various types of DCE-MRI data using various mathematical models. This flexibility would be especially advantageous when a single model is not optimal for all voxels in a given DCE-MRI dataset due to the presence of “kinetic heterogeneity” in malignant tumors [7, 20, 21]. If the fast GPU-based kinetic modeling approach proposed by this study can be perfected, one can construct an efficient DCE-MRI analysis method in which multiple model fittings are performed on each voxel’s data in order to find the best-fitting model that accurately represents the underlying physiology of that voxel. Results from this more thorough modeling process would not only provide better parameter estimates of the data, but would also allow the researchers to gain a deeper understanding of the pathology and the drug treatment in question.

## Supporting Information

S1 Program Main MATLAB program code of GPU-accelerated compartment model analysis. (M)

S2 Program A GPU-accelerated Nelder-Mead simplex-based minimization algorithm. (M)

S3 Program Extended Tofts model that used to depict contrast agent kinetics in tumor and other brain tissue. (M)

S1 Fig The *K* ^*trans*^ results of a single axial slice from DCE-MRI brain scans. The top row is from a pre-treatment scan and the bottom row is from a post-treatment scan. The left panel displays *K* ^*trans*^ values derived from the slices, and the right panel shows close-up *K* ^*trans*^ heat maps of the *ROI*.
(TIF)
