# Supplementary figures and images for: GPU-Accelerated Compartmental Modeling Analysis of DCE-MRI Data from Glioblastoma Patients Treated with Bevacizumab
Source: PLoS One. 2015 Mar 18;10(3):e0118421. doi: 10.1371/journal.pone.0118421 (PMC4364976; doi:10.1371/journal.pone.0118421)

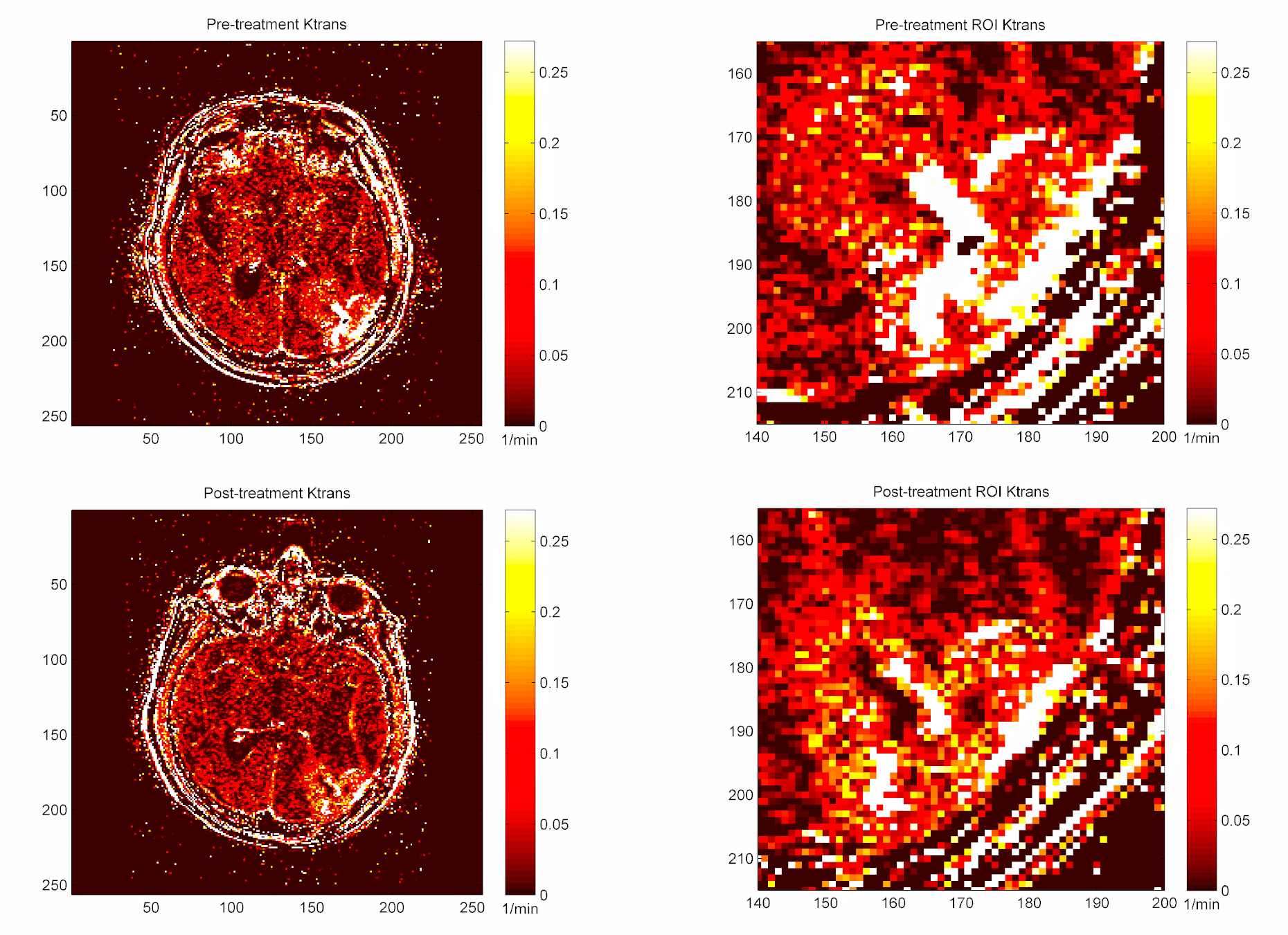

Supplement: S1 Fig — The top row is from a pre-treatment scan and the bottom row is from a post-treatment scan. The left panel displays K trans values derived from the slices, and the right panel shows close-up K trans heat maps of the ROI. (TIF) [file pone.0118421.s004.tif]
